# Case Teaching in Medicine

**Published:** 1906-04

**Authors:** 


CASE TEACHING IN MEDICINE. Exercises in Diagnosis, Prognosis and Treatment of Actual Cases. By Richard C. Cabot, M.D. (Harvard). 8vo. 220 pp. with space for notes. Introduction and indexes. There are 78 histories. Each is of special value in teaching and is followed by questions, answers, diagnosis, prognosis, treatment. From the students' edition answers are omitted and space left to be filled by the student himself. The book represents a plan that has been tried and is successful. D. C. Heath & Co., Publishers, 120 Boylston street, Boston. $1.50.

The plan of teaching medicine as described and arranged in this book has been tried by Dr. Cabot and some other eminent teachers, and found successful. The main object of the book is to aid the teacher in training his pupils to think clearly, cogently, and sensibly about the data gathered by physical examination. They are to be assisted in reasoning, for after the student has learned to open his eyes and see, he must learn to shut them and think. This book will undoubtedly prove of much value to both teachers and students. Dr. Cabot has brought forward a new and important method of using cases in teaching medicine, and we commend it heartily.

